# BRAF inhibitor candidate molecule usnic acid might use both intrinsic and extrinsic pathways of apoptosis

**DOI:** 10.55730/1300-0144.5890

**Published:** 2024-08-09

**Authors:** Burcu Pelin BÜYÜK, Demet CANSARAN DUMAN, Türker DUMAN

**Affiliations:** 1 Department of Biology, Faculty of Science, Ankara University, Ankara, Turkiye; 2 Biotechnology Institute, Ankara University, Ankara, Turkiye; 3 Rare Diseases Application and Research Center, Ankara University, Ankara, Turkiye

**Keywords:** Melanoma, usnic acid, apoptosis, caspases

## Abstract

**Background/aim:**

Melanoma is one of the most aggressive cancers and treatment methods commonly used for patients with skin cancer include checkpoint and BRAF/MEK inhibitors, traditional chemotherapy drugs, radiation, and adjuvant treatment methods. Due to the resistance and toxic effects that patients develop against the drugs, an effective treatment method has not been developed for melanoma yet. In this study we evaluated the anticancer effect of usnic acid (UA) on A-375 melanoma cells and human epidermal melanocytes using the xCELLigence real-time cell analysis system.

**Materials and methods:**

To determine the cell death pathway through which UA exerts its antiproliferative effect, its potential for apoptotic effects was investigated. Caspase-3 and caspase-9 enzyme assays and the expression analysis of 84 genes from the apoptosis pathway were carried out in UA-treated and nontreated A-375 cells.

**Results:**

UA was found to have an antiproliferative effect on A-375 cells while it did not have a cytotoxic effect on human epidermal melanocytes. UA treatment led to statistically significant increases in both caspase-3 and caspase-9 enzyme activities. Moreover, the expression levels of 61 genes (mainly proapoptotic genes) were increased and the expression levels of 23 genes (mainly antiapoptotic genes) were decreased in response to UA treatment. This effect might have developed through both the extrinsic and intrinsic apoptosis pathways; however, the extrinsic pathway was more pronounced.

**Conclusion:**

As a result of the obtained findings, it could be concluded that UA might be a promising candidate drug molecule for melanoma treatment in the future through topical application or encapsulation with nanocarriers.

## 1. Introduction

Usnic acid (UA) is a naturally occurring secondary metabolite of the fungal partner of lichen. It is known for its (+)-UA and (−)-UA enantiomers, which are distinguished by the orientation of the methyl group at position 9b [1]. Its dibenzofuran structure is what distinguishes UA, also known by the chemical name 2,6-diacetyl-7,9-dihydroxy-8,9b-dimethyl-1,3(2H,9bH)-dibenzofurandione. A major target for the pharmaceutical industry, UA possesses a variety of biological activities including antimicrobial, antiviral, antiprotozoal, antiproliferative, anticancer, antiinflammatory, antimetastatic, antiangiogenic, and analgesic activity [2]. Moreover, UA has been used as a component of various commercial products including mouthwashes, sunscreens, lotions, toothpastes, and deodorants [3]. The first study highlighting the anticancer potential of UA was conducted by Kupchan and Kopperman [4], who determined the effects of UA extracted from *Cladonia leptoclada* on lung cancer in vitro and in vivo. Since then, many studies have been performed on different cancer types to investigate the anticancer effects of UA [2,5,6,7]. However, UA’s mechanism of action is still not clear. According to some studies, it may involve inhibiting RNA transcription and DNA replication in tumor cells, which ultimately lowers the rate at which cancer cells proliferate or speeds up tumor cell apoptosis [4,6,7].

Melanoma, a type of skin cancer, results from the conversion of melanocytes, which produce melanin pigment and provide photoprotection for healthy cells, into malignant cells [8–10]. It can originate from pigment-producing cells in the meninges, gastrointestinal system, eyes, genitalia, or sinuses but UV damage to the skin is its most prevalent cause. Topical medication, surgery, radiation, immunotherapy, adjuvant therapy, sentinel lymph node biopsy, and chemotherapy are traditional treatment options for melanoma [11]. Chemotherapy is considered to be the most common treatment option for melanoma and 33 chemotherapy drugs have been approved by the US Food and Drug Administration (FDA) according to the most recent National Cancer Institute (NIH) list for melanoma treatment.^1^

Among the chemotherapy drugs included in the NIH list, dacarbazine is known as being the most effective drug, especially for treatment of advanced malignant melanoma. However, melanoma cells are resistant to traditional chemotherapeutic drugs due to the multifactorial nature of the disease [12]. For this reason, the therapeutic effectiveness of novel drug candidate molecules on melanoma has become a popular research topic [13]. Although numerous studies have revealed the anticancer effects of UA on different cancer types, relatively few studies have focused on melanoma [2,7,14,15]. Since UA has previously been shown to inhibit tumor cell proliferation through the induction of apoptosis and the inhibition of tumor angiogenesis, it is important to explore its antiproliferative potential against melanoma cells and thus fill the gap in the literature [16–18]. In some of the studies that focused on the roles of UA in the apoptosis pathway, it was declared that UA induced apoptosis via arrest of the G0/G1 or G2/M phase as a result of changes in the expression of some genes such as those of cyclin dependent kinases, CDK inhibitor proteins, and p53 mRNA [40,43,56]. On the other hand, a series of intricate and sophisticated molecular events are involved in the mechanisms of apoptosis. Therefore, more studies are needed to help researchers better understand the role of UA in apoptosis pathways.

Cell viability measurement is a critical application in cell biology and there are several conventional testing methods including the MTT (3-(4,5-dimethylthiazol-2-yl)-2,5-diphenyltetrazolium bromide) and trypan blue assays, which are colorimetric assays. With developments in technology, alternative methods have also been invented and began being used by researchers in the last decades [19–21]. The xCELLigence system (ACEA, Roche Diagnostics, Mannheim, Germany), which is based on biosensor technology, is one of these novel technologies with high sensitivity and specificity. It allows real-time monitoring of the cell index (CI) since it assesses the net cellular adhesion (focal adhesions) within an e-plate well [22]. The CI score constitutes important data from xCELLigence analysis because it shows the adhesion level of cells to the plate’s surface. It increases when the cells are present and growing, but the CI score will be zero if there are no adherent cells on the plate’s surface. In short, higher CI scores reflect greater amounts of adhesion. xCELLigence technology has been validated in many studies to analyze any reactions that cause changes in cell morphology, cell number, or cell movement [23].

In this study, we investigated the potential of UA in inhibiting melanoma development and progression through a focus on the apoptotic pathway. In this regard, the xCELLigence RTCA S16 Real-Time Cell Analysis System was used for the first time to determine the antiproliferative and cytotoxic effects of UA on A-375 melanoma cells compared to human epidermal melanocytes. Furthermore, this is the most comprehensive expression study conducted to date with the aim of measuring the mRNA levels of 84 genes in the apoptosis pathway in A-375 melanoma cells in response to UA administration.

## 2. Materials and methods

### 2.1. Cell culture conditions and UA treatment

Human epidermal melanocytes (Cat No. CC-2586) and A-375 melanoma cells (Cat No. CRL-1619) were purchased from the American Type Culture Collection (ATCC, Manassas, VA, USA). The A-375 melanoma cells were cultivated in Dulbecco’s minimum essential medium (DMEM) supplemented with 10% fetal bovine serum, 10,000 U/mL of 1% penicillin, and 10,000 μg/mL streptomycin (Cat. No. D6429, Sigma, St. Louis, MO, USA). Human epidermal melanocytes were cultured with the MGM-4 Melanocyte Growth Media-4 Bullet Kit (Cat. No. CC-3249, Lonza, Visp, Switzerland). Cells in T25 flasks were incubated in a CO_2_ incubator, which was set to 37 °C and 5% CO_2_ concentration. (+)-UA (>98% pure) was purchased from Santa Cruz Biotechnology (Cat. No. sc-204936A, Santa Cruz Biotechnology, Santa Cruz, CA, USA). A UA stock solution of 50 μM was prepared with DMSO (0.05%) and then diluted to different concentrations (1.56, 3.125, 6.25, 12.5, 25, and 50 μM) using DMEM. The same volume of 0.05% DMSO solution was added to the media of the control groups to keep a standard consistency. Established cell lines (i.e., human epidermal melanocytes and A-375 melanoma cells) that are commercially available (i.e., through the ATCC) do not require ethics committee approval to be procured or used.

### 2.2. xCELLigence assay

Cell proliferation was assessed with the xCELLigence RTCA S16 Real-Time Cell Analysis System (ACEA, Roche Diagnostics, Mannheim, Germany). A-375 melanoma cells (5 × 10^5^) and human epidermal melanocytes (5 × 10^5^) were seeded into e-plates and incubated using standard culture conditions. The xCELLigence RTCA station was placed into a CO_2_ incubator, which was set to 37 °C and 5% CO_2_ concentration. Following the growth of the cells for 24 h, cells were treated with different UA concentrations (1.56, 3.125, 6.25, 12.5, 25, and 50 μM). The CI and IC_50_ values were calculated using the xCELLigence RTCA software.

### 2.3. Exploring the morphology of cells in response to UA treatment

A-375 melanoma cells (5 × 10^5^) were seeded in a 6-well plate for cell imaging. After a 24-h incubation period, the IC_50_ concentration of UA was applied to A-375 melanoma cells and human epidermal melanocytes for 24 h. Inverted microscopy images were obtained at 10× magnification (TMS Inverted Phase Contrast Microscope, Nikon, Tokyo, Japan).

### 2.4. Caspase-3 and caspase-9 enzyme activity assays

Enzyme activities were measured using caspase-3 and caspase-9 colorimetric kits (Sigma, USA) in compliance with the manufacturer’s guidelines. In a 6-well cell plate, 5 × 10^5^ cells were planted per well. The cells were then incubated for 24 h at 37 °C with 5% CO_2_. Cells were exposed to UA at the IC_50_ concentration and then incubated for 24 h. On a 96-well plate, a reaction mixture (total volume: 100 μL) including 5 μL of cell lysate and 10 μL of caspase-3 substrate Ac-DEVD-pNA (2 mM) was utilized. To account for nonspecific hydrolysis of the substrate, conditions were generated in a control reaction mixture assay buffer containing 10 μL of the specific caspase-3 inhibitor Ac-DEVD-CHO (200 M) and 5 μL of cell lysate. After both combinations were incubated overnight at 37 °C, the absorbance (OD) values were measured at 405 nm with an Infinite M Plex multimode plate reader (Tecan, Grödig, Austria), and caspase-3 and caspase-9 activities were then calculated as percentages.

### 2.5. RT-qPCR analysis

Using the TRIzol reagent (Thermo Fisher Scientific, Waltham, MA, USA) following the manufacturer’s instructions, total RNA was extracted from cells that were exposed to the IC_50_ concentration of UA. Utilizing NanoDrop (NanoDrop Lite Spectrophotometer, Thermo Fisher Scientific, USA), the concentration and purity of the isolated RNAs were assessed. The NG dART RT Kit (EURx, Gdansk, Poland) was used for cDNA synthesis in accordance with the manufacturer’s instructions. The commercial RT^2^ Profiler PCR Array (Cat. No. PAHS-012Z, QIAGEN, Hilden, Germany) was used to evaluate the effects of UA on the mRNA levels of 84 key genes involved in the apoptosis pathway in A-375 melanoma cells. RT-qPCR data normalization was done using the RT^2^ Profiler PCR data analysis tool included with the RT^2^ Profiler PCR Array (https://dataanalysis2.qiagen.com) for the reference genes (*ACTB*, *B2M*, *GAPDH*, *HPRT1*, and *RPLP0*).

### 2.6. Statistical analysis

Statistical analyses were performed using Prism 9 software (GraphPad, Boston, MA, USA). Three replicates of the RT-qPCR experiments were performed for each condition. One-way analysis of variance (ANOVA) followed by the Tukey test was used for multiple comparisons. The significance level was accepted as p < 0.05.

## 3. Results

### 3.1. Determination of antiproliferative and cytotoxic effects of UA

**Figure 1:**
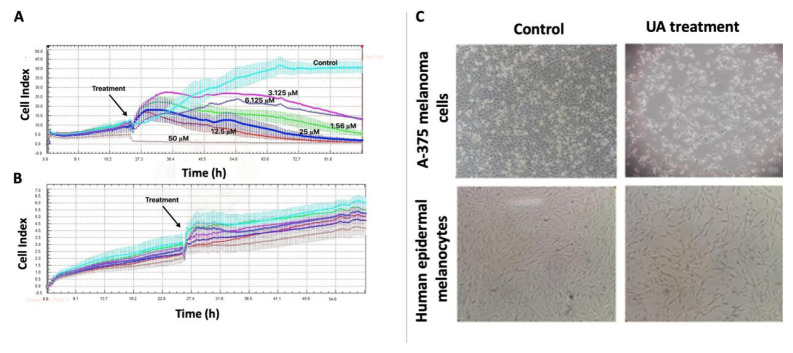
**a)** Normalized cell index graph for A-375 melanoma cells and **b)** human epidermal melanocytes treated with different concentrations of UA (0, 1.56, 3.125, 6.25, 12.5, 25.0, and 50.0 μM) (turquoise: plain medium (control); green: 1.56 μM; pink: 3.125 μM; purple: 6.25 μM; red: 12.5 μM; blue: 25 μM; brown: 50 μM). **c**) Effects of the IC_50_ concentration of UA treatment on A-375 melanoma cells and human epidermal melanocytes in comparison to control cells. Photos were taken using an inverted microscope at 10× magnification.

Different UA concentrations (1.56, 3.125, 6.25, 12.5, 25, and 50 μM) were applied to human epidermal melanocytes and A-375 melanoma cells in this study. Comparisons were made between UA-treated and control group cells. The control groups of both cell lines were cultured in media containing the same volume of 0.05% DMSO as in the UA treatment group. The cytotoxic and antiproliferative effects of UA on epidermal melanocytes and A-375 melanoma cells were analyzed using the xCELLigence RTCA S16 system. CI versus time (h) curves for human epidermal melanocytes and A-375 melanoma cells as obtained from the xCELLigence system with different concentrations of UA are shown in Figures 1a and 1b. The responses of the cell lines to UA treatment were different in terms of time (h) and concentration. For instance, in A-375 melanoma cells, the CI value was reduced following the changing of media including different concentrations of UA at 24 h, suggesting that the proliferation of A-375 cells was affected by UA treatment (Figure 1a).

According to the results provided by the xCELLigence software, no cytotoxicity was observed in human epidermal melanocytes treated with 1.56, 3.125, 6.25, 12.5, and 25 μM concentrations of UA. However, UA was found to have a concentration-dependent and time-dependent cytotoxic effect on human epidermal melanocytes at 50 μM and 74 h (Figure 1b).

The antiproliferative effect on A-375 melanoma cells after treatment with 1.56, 3.125, 6.25, 12.5, and 25 μM concentrations of UA was also observed. Using the RTCA software, the IC_50_ concentration of UA against A-375 melanoma cells was found to be 20 μM at 45 h (p < 0.05) (Figure 1a). UA had a concentration-dependent and time-dependent cytotoxic effect on A-375 melanoma cells at 50 μM and 26 h (Figure 1a).

### 3.2. Changes in cell morphology of A-375 melanoma cells and human epidermal melanocytes in response to UA

Human epidermal melanocytes and A-375 melanoma cells were examined using inverted microscopy to identify morphological changes following UA treatment at the IC_50_ concentration. A-375 melanoma cells were found to show different morphology compared to control cells following UA administration. However, limited or essentially no morphological differences were observed in human epidermal melanocytes in response to UA treatment (Figure 1c).

### 3.3. Changes in caspase-3 and -9 enzyme activities in response to UA

**Figure 2:**
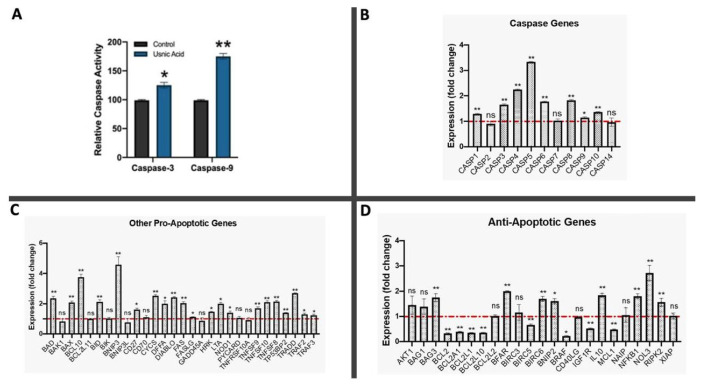
**a)** Changes in caspase-3 and caspase-9 enzyme activities in response to UA treatment in A-375 melanoma cells. **b–d)** Fold changes in the expression levels of caspase genes in A-375 melanoma cells in response to usnic acid treatment compared to the control (*: p<0.05, **: p<0.01, ns: not significant).

The effects of UA on caspase-3 (executer) and caspase-9 (initiator) enzyme activities in A-375 melanoma cells were evaluated. The changes in caspase-3 and caspase-9 enzyme activities were compared between control and UA-treated A-375 melanoma cells. Statistically significant increases were detected in the activities of both enzymes in response to UA treatment. As a result, 1.75- and 1.3-fold increases were observed in caspase-3 and caspase-9 enzyme activities compared to the control, respectively (Figure 2a).

### 3.4. Correlation between mRNA levels of genes involved in the apoptotic pathway and UA treatment

The RT^2^ Profiler PCR Array was used to evaluate the effects of UA on mRNA levels of 84 key genes involved in the apoptosis pathway in A-375 melanoma cells. In addition, 5 reference genes (*ACTB*, *B2M*, *GAPDH*, *HPRT1*, and *RPLP0*) were used to normalize raw RT-qPCR data. The descriptive information of the studied genes (84 + 5 genes) is presented in the Supplementary Table. Initially, RT-qPCR analysis of reference genes in control and UA-treated A-375 melanoma cells was performed. The geometric mean of the cycle threshold (CT) values obtained from those reference genes was then calculated and the normalization of qPCR data was carried out over that geometric mean.

The expression levels of caspase genes were evaluated and the majority were found to be upregulated in A-375 melanoma cells in response to UA treatment. The *Caspase-5*, *Caspase-4*, *Caspase-8*, *Caspase-6*, *Caspase-3*, *Caspase-10*, *Caspase-1*, and *Caspase-9* genes showed the highest expression levels among all studied caspase genes (Figure 2b). On the other hand, the *Caspase-14*, *Caspase-7*, and *Caspase-2* genes revealed decreased mRNA levels in response to UA treatment in A-375 melanoma cells, although these decreases were statistically insignificant (p < 0.05) (Figure 2b).

The mRNA levels of proapoptotic genes excluding caspases were measured. The *BNIP3* gene was found to have the highest expression level (4.76-fold) in A-375 melanoma cells in response to UA treatment. *BCL10* (3.84-fold), *TRADD* (2.71-fold), *CYCS* (2.51-fold), *DIABLO* (2.43-fold), *BAD* (2.36-fold), *BID* (2.17-fold), *TNFSF8* (2.13-fold), *TNFSF10* (2.11-fold), *BAX* (2.03-fold), and *FAS* (2.01-fold) were also proapoptotic genes highly upregulated in response to UA treatment in A-375 melanoma cells (Figure 2c). There were few downregulated genes in this group, including *BAK1*, *BNIP3L*, *GADD45A*, and *TNFRSF10A*. However, these downregulations were found to be statistically insignificant (p < 0.05) (Figure 2c).

In this study, 23 antiapoptotic genes that play crucial roles in the apoptosis pathway were also studied. Eight of the 23 antiapoptotic genes were found to be upregulated in response to UA treatment compared to the control (p < 0.05) (Figure 2d). These upregulated genes were *NOL3* (2.75-fold), *BFAR* (1.99-fold), *IL10* (1.80-fold), *NFKB1* (1.80-fold), *BIRC6* (1.79-fold), *BAG3* (1.73-fold), *BNIP2* (1.61-fold), and *RIPK* (1.59-fold). On the other hand, *BIRC5*, *IGFR1*, *MCL1*, *BRAF*, *BCL2A1*, *BCL2L10*, *BCL2L1*, and *BCL2* were downregulated following UA treatment in A-375 melanoma cells (p < 0.05) (Figure 2d).

**Figure 3:**
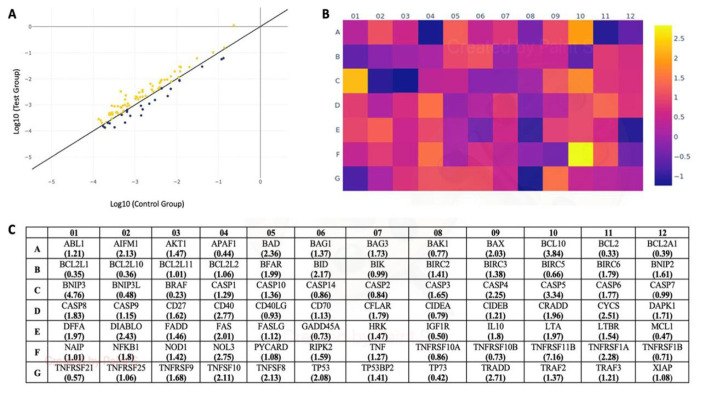
**a)** Scatter plot graph shows the number of differentially expressed genes. Yellow and black dots indicate upregulated and downregulated genes in response to UA treatment, respectively. **b)** Heat map shows the differentially expressed genes in A-375 melanoma cells in response to UA. **c)** Gene expression fold changes in A-375 melanoma cells in response to UA.

Death domain receptors, which include a segment of about 80 amino acids in length known as the death domain (DD), are crucial components of the apoptosis process. They trigger different proteins that mediate programmed cell death when activated by a ligand. In this study, the gene expression levels of four death domain receptors with proapoptotic properties were investigated (Figures 3a–3c). It was discovered that TNF death domain receptor genes including *FADD* and *CRADD* showed considerably higher expression levels in response to UA treatment compared to the control group (p < 0.05). On the other hand, it was seen that *TNFRSF10B* was downregulated compared to the control, but this downregulation was statistically insignificant (p < 0.05) (Figure 3c).

Some of the key genes involved in the apoptosis pathway are known to be associated with DNA damage and repair mechanisms in the cell [24]. In this study, the gene expression levels of such genes, including *ABL1*, *CIDEA*, *CIDEB*, *TP53*, and *TP73*, were analyzed. Three of them (*ABL1*, *CIDEB*, and *TP53*) were found to be upregulated, and *TP53* (2.08-fold) showed the most dramatic upregulation response to UA treatment in A-375 melanoma cells. The *CIDEA* and *TP73* genes were downregulated in response to UA treatment, but only the downregulation of *TP73* was statistically significant (p < 0.05) (Figure 3c).

The expression levels of *CFLAR* (*CASPER*), *DAPK1*, and *TNFRSF25* (*DR3*), as genes responsible for extracellular apoptotic signals, were also studied (Figure 3c). All of them were found to be upregulated in response to UA treatment. However, only the upregulation of *TNFRSF25* was statistically insignificant (Figure 3c).

The mRNA levels of some other apoptosis-related genes (*AIFM1*, *APAF1*, *BIRC2*, *CD40*, *LTBR*, *TNFRS11B*, *TNFRSF1A*, *TNFRSF1B*, *TNFRSF21*, and *TRAF*) were also studied, although they were not listed in the above categories according to the guidelines of the system’s manufacturer (Figures 3b and 3c). Among these genes, *TNFRSF11B* (7.16-fold), *CD40* (2.77-fold), *TNFRSF1A* (2.28-fold), *AIFM1* (2.13-fold), and *LTBR* (1.54-fold) were the most dramatically upregulated in response to UA treatment. On the other hand, *APAF1*, *TNFRSF21*, and *TNFRSF1B* were downregulated after UA treatment, although these downregulations were only statistically significant for *APAF1* and *TNFRSF21* (p < 0.05) (Figure 3c).

## 4. Discussion

Chemotherapy is one of the most effective treatment options for most cancer types, but melanoma cells were shown to be resistant to standard chemotherapeutics [13]. Therefore, novel drug candidate molecules including secondary metabolites derived from different organisms have been investigated to find better treatment alternatives for melanoma. Cell death pathways could control cancer cells, which can evade immune surveillance and survive under adverse conditions to promote melanoma growth [25]. Apoptosis, or programmed cell death, is a significant cell death mechanism that acts as a natural barrier to stop human melanoma cells from surviving and continuously proliferating [26]. However, cancer cells could still escape apoptosis and this may lead to uncontrolled proliferation of melanoma cells, which then promotes carcinogenesis [27,28].

Recent studies have focused on the medicinal potential of lichens because of the unique properties originating from their secondary metabolites [29–31]. In particular, the use of UA, which is a lichen secondary metabolite, has drawn significant interest in recent studies because of its antibacterial, antiviral, antiinflammatory, antioxidant, curative, and anticancer characteristics [30,32]. However, alongside its positive impacts, usnic acid might be toxic to cells in general and to the liver according to studies carried out in vitro and in vivo [33]. Moreira et al. [34] investigated the hepatoxicity of UA in rats and found that it uncoupled oxidative phosphorylation in cells at concentrations lower than 2.5 μM, which may lead to the inhibition of ATP synthesis. According to the same study [34], UA might lead to critical problems in cells by blocking electron transfers in the mitochondria at high concentrations. Moreover, 21 cases associated with UA use and severe liver responses such as liver failure and hepatic necrosis have been documented by the FDA [35]. However, several studies suggested that the hepatotoxicity of UA may be dose-dependent and limited to specific doses. Consequently, limits have been imposed on the consumption of UA or products containing UA, severely restricting their application in therapeutic settings [36]. These considerations have prompted research to reduce the liver damage caused by UA while maintaining its therapeutic effects. Recently, there has been new evidence that encapsulating UA in appropriate polymer nanocarriers may reduce its hepatotoxicity [37–39].

For many reasons, it is important to identify the application dose and application method of UA for possible cancer treatment strategies in the future. Therefore, in this study, we initially aimed to determine the cytotoxic concentrations of UA against human epidermal melanocytes and A-375 cells. No cytotoxicity was observed in human epidermal melanocytes treated with UA at concentrations of 1.56, 3.125, 6.25, 12.5, and 25 μM (Figure 1b). The IC_50_ value of UA for melanoma treatment was determined to be 20 μM in this study and assays were conducted using that concentration of UA since it was also within the range determined to be safe for human epidermal melanocytes.

A range of cancer types, including stomach, hepatic, lung, breast, and prostate cancer, have been studied for identifying the antiproliferative effects of UA [40,41]. Wu et al. [42] observed that UA at the IC_50_ dose (21–68 μM) inhibited the growth of lung cancer cells from the SPC-A-1, 95D, and SK-LU-1 cell lines. Similarly, in the present study, UA was found to inhibit the growth of A-375 melanoma cells. Moreover, the IC_50_ value for UA treatment of lung cancer reported by Wu et al. [42] was similar to that obtained for A-375 melanoma cells in our study. In another study, Petrová et al. [43] showed that UA treatment at the IC_50_ concentration (33.57 μM) inhibited neoangiogenesis, which is a characteristic of growing solid tumor tissue, in human umbilical vein endothelial cells. The results obtained by Petrová et al. [43] are parallel to the findings of the present study, as UA was shown to inhibit the development and progression of A-375 melanoma cells. Çolak et al. [44] studied the effects of UA treatment on ovarian cancer and found that UA achieved concentration- and time-dependent inhibition of ovarian cancer cell proliferation and induced programmed cell death, similar to the findings obtained in our study. Based on the type and characteristics of the cancer cells, different UA treatment concentrations have been found to have different effects on cells [45].

In this study, the anticancer activity of UA on A-375 melanoma cells was investigated. The mRNA analysis of 84 apoptosis genes was performed following the determination of the IC_50_ value of UA to evaluate the UA-induced apoptosis pathway. Consequently, it was shown that the expression level of 61 of 84 genes involved in the apoptotic pathway increased in A-375 melanoma cells treated with UA. The total number of genes that were downregulated was 23 in A-375 melanoma cells treated with the IC_50_ dose of UA. In light of these findings, it was concluded that most genes playing roles in the apoptosis pathway were induced by UA treatment and these were mainly proapoptotic genes. In a previous study by Yangın et al. [13], vulpinic acid, which is another secondary metabolite of lichens, was also found to upregulate a significant number of genes in the apoptotic pathway in melanoma cells, similar to our findings. Çolak et al. [44] also declared that UA treatment led to the upregulation of most apoptotic genes, especially caspases, in ovarian cancer cells.

The expression levels of 11 caspase genes, known as the processors of apoptosis, were also evaluated in this study. It was seen that eight of them were significantly upregulated in response to UA treatment compared to the control, while statistically insignificant decreases were observed for the remaining caspase genes (Figure 2b). From this point of view, it was clearly understood that UA led to increases in the expression levels of most caspase genes in A-375 melanoma cells. Caspases are a class of endoprotease enzymes that regulate cell death [46]. They bind to substrates and cause them to become active, but in some processes caspases can also inactivate substrates. However, by creating potent signaling molecules, caspase enzymes can contribute to inflammation and apoptosis [46].

Caspases are classified into a number of categories based on how they contribute to apoptosis and inflammation. *Caspase-1*, *-4*, *-5*, and *-12* are engaged in inflammation while *Caspase-3*, *-6, -7*, *-8*, and -*9* are involved in apoptotic processes. Caspase enzymes that are engaged in apoptotic processes are further divided into two subclasses of executor (caspase-3, -6, and -7) and initiator (caspase-8 and -9) caspase enzymes. The apoptotic process progresses gradually following the activation of executioner caspases by initiator caspases. Studying the caspase enzymes that are directly associated with the apoptosis pathway is crucial for examining drug candidate molecules [47]. The expression levels of caspase genes were compared in this study and the order of the genes from highest to lowest expression level under UA treatment was as follows: *Caspase-5*, *-4*, *-8*, *-6*, *-3*, *-10*, *-1*, and *-9* (Figure 2b). However, the alterations in the expression levels of *Caspase-14*, *Caspase-7*, and *Caspase-2* in response to UA treatment were statistically insignificant. *Caspase-8*, among the known initiator caspase genes, and *Caspase-3*, one of the executioner caspases, showed higher expression levels than other caspases (Figure 2b). This may indicate that UA would use the extrinsic pathway of apoptosis, as previously suggested by Wu et al. [48].

The expression levels of many proapoptotic genes associated with the extrinsic apoptosis pathway (*FAS*, *FASLG*, *TNFRSF11B*, *TNFRSF10*, *TNFRSF1A*, *TNF*, *FADD*, *TNFRSF9*, *TRAD*, *TRAF2*, *TRAF3*, *NFKB1*, *CD27*, *CD40*, and *TNFRSF8*) were also upregulated in response to UA treatment in this study (Figures 2c and 3c). The proapoptotic *Caspase-10* gene, a homolog of *Caspase-8*, which is an initiator caspase, was also found to be significantly upregulated in response to UA treatment [49]. On the other hand, alterations in the expressions of genes of the TNF family including *TNFRSF25*, *TNFRSF1B*, *CD40LG*, and *CD70* were found to be statistically insignificant compared to the control (Figure 2c). The decrease in the expression of proapoptotic *TNFRSF21* in response to UA treatment, with this gene playing a role in the extrinsic apoptosis pathway, and the increase in the expression level of the antiapoptotic *CFLAR* gene are not enough to refute this inference because the balance between proapoptotic and antiapoptotic genes in apoptosis determines the fate of cells [50].

Inhibitor of apoptosis proteins (IAPs) are negative regulators of both caspases and cell death [51]. In this study, the transcript levels of IAP genes including *NAIP* (*BIRC1*), *BIRC2* (*CIAP1*), *BIRC3* (*CIAP2*), *BIRC5* (*Survivin*), *BIRC6* (*Apollon*), and *XIAP* (*BIRC4*) in UA-treated A-375 melanoma cells were investigated. A statistically significant increase was only observed in the expression of *BIRC6*, which is an antiapoptotic gene. This result might indicate that UA does not increase the expression levels of IAPs with one exception, which involves a negative regulator of apoptosis. On the other hand, the expression of *BIRC5* (*Survivin*), an antiapoptotic IAP gene, was significantly decreased compared to the control. It was thought that this decrease in *BIRC5* could be related to the high expression of the *p53* gene (2.08-fold) determined in the A-375 melanoma cells in response to UA treatment in this study. In previous studies, it was stated that there is a correlation between p53 and survivin protein expression, and that p53 protein reduces the antiapoptotic effect of survivin protein by suppressing it [43,52].

The tumor suppressor gene *TP53* is known to be mutated in approximately 50% of human cancers [52]. In addition to its function in tumor suppression, *p53* also plays an important role in the response of malignant and nontransformed cells to many anticancer therapeutics, particularly those that cause DNA damage. *p53* forms a homotetrameric transcription factor that has been reported to directly regulate approximately 500 target genes, thereby controlling various processes such as cell cycle arrest, cell senescence, DNA repair, metabolic adaptation, and cell death. The *p53* gene is known to inhibit tumor development through the induction of apoptosis [52]. In this study, it was determined that the *p53* gene was upregulated (2.08-fold) in A-375 melanoma cells in response to UA treatment. Additionally, the expression of *TP53BP2*, which is responsible for the positive regulation of the p53 protein, was found to increase compared to the control. This showed that tumors might be suppressed in A-375 melanoma cells with UA treatment, in agreement with the findings of Huo et al. [53].

The *p73* gene, which is known as a *p53* homolog due to their structural similarity, was also evaluated in this study and it was determined that there was a decrease (2.36-fold) in its expression level compared to the control in response to UA treatment. However, the functioning mechanism of *p73* is not as clear as that of *p53* and it is known that decreases in *p73* do not affect *p53* activity [54]. There is still uncertainty in the literature about the role of *p73* in apoptosis regarding whether it is proapoptotic or antiapoptotic [53,55].

Because of their multiple functions in cancer, BCL-2 family proteins, which can be antiapoptotic or proapoptotic, have become interesting targets for anticancer drugs. For this reason, the transcript levels of BCL-2 family genes in response to UA treatment in A-375 melanoma cells were also investigated. It was seen that the expression levels of antiapoptotic genes including *BCL2*, *BCL2L1*, *BCL2A1*, and *BCL2L10* were significantly downregulated compared to the control in response to UA treatment. Moreover, it was determined that the *MCL1* gene, which is another antiapoptotic gene of the BCL-2 family, was significantly downregulated, while the expression level of *BCL2L2* was not changed under UA treatment. On the other hand, when the proapoptotic BCL-2 family members were evaluated, it was determined that *BAX*, *BID*, *BAD*, *HRK*, *BNIP3*, and *BCL10* were significantly upregulated, while the expression level of *BNIP3L* decreased in response to UA treatment. Since the expression changes in the *BAK1*, *BCL2L11*, and *BIK* genes were not statistically significant, the data were not reported.

The BRAF protein promotes cell division through the signals it sends into cells and regulates apoptosis, among other functions that contribute to cell growth. Noncancerous cells can develop into cancerous cells when there is a mutation in the *BRAF* gene. Although *BRAF* mutations occur in different types of cancer, they are more frequently detected in melanoma [56]. In this study, it was observed that the expression of the *BRAF* gene in A-375 melanoma cells, which is known to have the *BRAF*-V600E mutation that causes skin cancer, was significantly inhibited by UA treatment. Therefore, UA might cause the suppression of cancer cell proliferation by inhibiting the expression of the *BRAF* gene carrying the *BRAF*-V600E mutation and by upregulating caspase genes, which are the processors of apoptosis.

Other studied genes that play a role in the intrinsic pathway of apoptosis included *APAF-1*, *Cytc* (responsible for cytochrome release), and *Caspase-9* in this study. It was observed that *Cytc* and *Caspase-9* were upregulated and *APAF-1* was downregulated in response to UA treatment. The proteins encoded by these three genes (*APAF-1*, *Cytc*, and *Caspase-9*) form the apoptosome complex and the apoptosis process starts following the activation of the caspase-3, -6, and -7 enzymes. In this study, it was found that the gene expression levels of *Caspase-3* and *Caspase-6* increased in response to UA treatment. Accordingly, we can suggest that UA treatment could also activate the internal mitochondrial pathway of apoptosis in malignant melanoma cells.

Evaluations of the effects of UA on cancer cells have revealed findings that UA may influence both the intrinsic and extrinsic pathways of apoptosis. Bačkorová et al. [56] stated that UA had an apoptosis-inducing effect on HT-29 colorectal adenocarcinoma and A2780 ovarian cancer cell lines, and that effect might be mediated by the mitochondrial pathway. Song et al. [5] further reported that UA induced apoptosis in the Bcap-37 breast cancer cell line using the mitochondrial pathway. Similarly, Geng et al. [57] determined that the apoptosis-inducing effect of UA on gastric cancer cells was mediated via the mitochondrial pathway. Çolak et al. [44] reported that UA induced apoptosis in ovarian cancer cells and that this occurred through the extrinsic pathway. In light of the data obtained in the present study, we concluded that UA induced apoptosis in A-375 melanoma cells, mainly by using the extrinsic apoptosis pathway. However, the regulation of the intrinsic pathway might also contribute to the induction of UA-mediated apoptosis according to our findings.

In the recent study by Galanty et al. [7] on UA and melanoma, the effects of the (+)-UA and (−)-UA enantiomers derived from lichen species *Cladonia arbuscula* and *C. uncialis* were tested on different melanoma cell lines including HTB140, A375, and WM793. The authors stated that (+)-UA and (−)-UA had a potential anticancer effect against melanoma and that (+)-UA could particularly be evaluated in further studies to understand its mechanism of action against melanoma because it was found to be safer than (−)-UA in toxicity studies. In our study, in contrast to the study conducted by Galanty et al. [7], we used commercial (+)-UA with high purity and its antiproliferative effect against melanoma cells was analyzed using the xCELLigence RTCA S16 Real Time Cell Analysis System for the first time. Additionally, we analyzed the mRNA levels of a significant number of genes involved in the apoptosis pathway to understand the mechanism of action of UA against A-375 melanoma cells.

In conclusion, this study aimed to evaluate the effects of UA, a lichen secondary metabolite, on human epidermal melanocytes and A-375 melanoma cells to determine its potential for inhibiting the proliferation of skin cancer cells. According to the results obtained, UA had an antiproliferative effect on A-375 melanoma cells and did not have a cytotoxic effect on melanocyte cells. Changes in the expression profiles of 84 genes related to apoptosis were also evaluated and it was determined that UA might have anticancer effects by inducing apoptosis in A-375 melanoma cells. This effect might be achieved by using both extrinsic and intrinsic pathways of apoptosis, but according to the mRNA profile, the extrinsic pathway was more pronounced. The results obtained from this study might indicate that UA could be used as a potential drug molecule candidate in the treatment of melanoma. However, there might be different alternatives for its delivery methods to be considered in future studies. The topical application of UA might be particularly suitable for early-stage melanoma, as it can be applied directly to the skin to exert a localized effect and minimize systemic side effects. Nanocapsules prepared using nanotechnology could help UA penetrate into deeper layers of the skin. Systemic administration may be considered for advanced cases of melanoma, but appropriate carrier systems are needed to reduce the systemic toxicity of UA and ensure its selective distribution. Lipid-based or nanoparticle-based carriers can increase the bioavailability of UA, enabling it to reach target cells with controlled release. Dosage optimization is critical to ensure that UA kills cancer cells without harming normal cells. In preclinical and clinical studies, the optimal dosage should be determined by evaluating the efficacy and safety of UA and adjustments should be made according to patients’ responses. Given that UA alters apoptotic gene expression and increases caspase-3 and caspase-9 enzyme activities, the dosage should be optimized to target those apoptotic pathways. Furthermore, the combination of UA with chemotherapy drugs or BRAF/MEK inhibitors may produce a synergistic effect, leading to higher treatment success at lower doses. There is a need for studies on the clinical use of UA and its effectiveness at the protein level. In this context, it is important for us and other researchers to conduct future research on these topics.

## Supplementary Materials

**Supplementary Table:** Gene table: RT^2^ Profiler PCR Array (QIAGEN, Cat. No. 330231 PAHS-012ZA).

Plate Position	UniGene	GenBank	Symbol	Description
A01	Hs.431048	NM_005157	ABL1	C-abl oncogene 1, non-receptor tyrosine kinase
A02	Hs.424932	NM_004208	AIFM1	Apoptosis-inducing factor, mitochondrion-associated, 1
A03	Hs.525622	NM_005163	AKT1	V-akt murine thymoma viral oncogene homolog 1
A04	Hs.728891	NM_001160	APAF1	Apoptotic peptidase activating factor 1
A05	Hs.370254	NM_004322	BAD	BCL2-associated agonist of cell death
A06	Hs.377484	NM_004323	BAG1	BCL2-associated athanogene
A07	Hs.523309	NM_004281	BAG3	BCL2-associated athanogene 3
A08	Hs.485139	NM_001188	BAK1	BCL2-antagonist/killer 1
A09	Hs.624291	NM_004324	BAX	BCL2-associated X protein
A10	Hs.193516	NM_003921	BCL10	B-cell CLL/lymphoma 10
A11	Hs.150749	NM_000633	BCL2	B-cell CLL/lymphoma 2
A12	Hs.227817	NM_004049	BCL2A1	BCL2-related protein A1
B01	Hs.516966	NM_138578	BCL2L1	BCL2-like 1
B02	Hs.283672	NM_020396	BCL2L10	BCL2-like 10 (apoptosis facilitator)
B03	Hs.469658	NM_006538	BCL2L11	BCL2-like 11 (apoptosis facilitator)
B04	Hs.410026	NM_004050	BCL2L2	BCL2-like 2
B05	Hs.435556	NM_016561	BFAR	Bifunctional apoptosis regulator
B06	Hs.591054	NM_001196	BID	BH3 interacting domain death agonist
B07	Hs.475055	NM_001197	BIK	BCL2-interacting killer (apoptosis-inducing)
B08	Hs.696238	NM_001166	BIRC2	Baculoviral IAP repeat containing 2
B09	Hs.127799	NM_001165	BIRC3	Baculoviral IAP repeat containing 3
B10	Hs.728893	NM_001168	BIRC5	Baculoviral IAP repeat containing 5
B11	Hs.150107	NM_016252	BIRC6	Baculoviral IAP repeat containing 6
B12	Hs.646490	NM_004330	BNIP2	BCL2/adenovirus E1B 19kDa interacting protein 2
C01	Hs.144873	NM_004052	BNIP3	BCL2/adenovirus E1B 19kDa interacting protein 3
C02	Hs.131226	NM_004331	BNIP3L	BCL2/adenovirus E1B 19kDa interacting protein 3-like
C03	Hs.550061	NM_004333	BRAF	V-raf murine sarcoma viral oncogene homolog B1
C04	Hs.2490	NM_033292	CASP1	Caspase 1, apoptosis-related cysteine peptidase (interleukin 1, beta, convertase)
C05	Hs.5353	NM_001230	CASP10	Caspase 10, apoptosis-related cysteine peptidase
C06	Hs.466057	NM_012114	CASP14	Caspase 14, apoptosis-related cysteine peptidase
C07	Hs.368982	NM_032982	CASP2	Caspase 2, apoptosis-related cysteine peptidase
C08	Hs.141125	NM_004346	CASP3	Caspase 3, apoptosis-related cysteine peptidase
C09	Hs.138378	NM_001225	CASP4	Caspase 4, apoptosis-related cysteine peptidase
C10	Hs.213327	NM_004347	CASP5	Caspase 5, apoptosis-related cysteine peptidase
C11	Hs.654616	NM_032992	CASP6	Caspase 6, apoptosis-related cysteine peptidase
C12	Hs.9216	NM_001227	CASP7	Caspase 7, apoptosis-related cysteine peptidase
D01	Hs.599762	NM_001228	CASP8	Caspase 8, apoptosis-related cysteine peptidase
D02	Hs.329502	NM_001229	CASP9	Caspase 9, apoptosis-related cysteine peptidase
D03	Hs.355307	NM_001242	CD27	CD27 molecule
D04	Hs.472860	NM_001250	CD40	CD40 molecule, TNF receptor superfamily member 5
D05	Hs.592244	NM_000074	CD40LG	CD40 ligand
D06	Hs.501497	NM_001252	CD70	CD70 molecule
D07	Hs.390736	NM_003879	CFLAR	CASP8 and FADD-like apoptosis regulator
D08	Hs.249129	NM_001279	CIDEA	Cell death-inducing DFFA-like effector a
D09	Hs.642693	NM_014430	CIDEB	Cell death-inducing DFFA-like effector b
D10	Hs.38533	NM_003805	CRADD	CASP2 and RIPK1 domain containing adaptor with death domain
D11	Hs.437060	NM_018947	CYCS	Cytochrome c, somatic
D12	Hs.380277	NM_004938	DAPK1	Death-associated protein kinase 1
E01	Hs.484782	NM_004401	DFFA	DNA fragmentation factor, 45kDa, alpha polypeptide
E02	Hs.169611	NM_019887	DIABLO	Diablo, IAP-binding mitochondrial protein
E03	Hs.86131	NM_003824	FADD	Fas (TNFRSF6)-associated via death domain
E04	Hs.244139	NM_000043	FAS	Fas (TNF receptor superfamily, member 6)
E05	Hs.2007	NM_000639	FASLG	Fas ligand (TNF superfamily, member 6)
E06	Hs.80409	NM_001924	GADD45A	Growth arrest and DNA-damage-inducible, alpha
E07	Hs.87247	NM_003806	HRK	Harakiri, BCL2 interacting protein (contains only BH3 domain)
E08	Hs.643120	NM_000875	IGF1R	Insulin-like growth factor 1 receptor
E09	Hs.193717	NM_000572	IL10	Interleukin 10
E10	Hs.36	NM_000595	LTA	Lymphotoxin alpha (TNF superfamily, member 1)
E11	Hs.1116	NM_002342	LTBR	Lymphotoxin beta receptor (TNFR superfamily, member 3)
E12	Hs.632486	NM_021960	MCL1	Myeloid cell leukemia sequence 1 (BCL2-related)
F01	Hs.710305	NM_004536	NAIP	NLR family, apoptosis inhibitory protein
F02	Hs.654408	NM_003998	NFKB1	Nuclear factor of kappa light polypeptide gene enhancer in B-cells 1
F03	Hs.405153	NM_006092	NOD1	Nucleotide-binding oligomerization domain containing 1
F04	Hs.513667	NM_003946	NOL3	Nucleolar protein 3 (apoptosis repressor with CARD domain)
F05	Hs.499094	NM_013258	PYCARD	PYD and CARD domain containing
F06	Hs.103755	NM_003821	RIPK2	Receptor-interacting serine-threonine kinase 2
F07	Hs.241570	NM_000594	TNF	Tumor necrosis factor
F08	Hs.591834	NM_003844	TNFRSF10A	Tumor necrosis factor receptor superfamily, member 10a
F09	Hs.521456	NM_003842	TNFRSF10B	Tumor necrosis factor receptor superfamily, member 10b
F10	Hs.81791	NM_002546	TNFRSF11B	Tumor necrosis factor receptor superfamily, member 11b
F11	Hs.279594	NM_001065	TNFRSF1A	Tumor necrosis factor receptor superfamily, member 1A
F12	Hs.256278	NM_001066	TNFRSF1B	Tumor necrosis factor receptor superfamily, member 1B
G01	Hs.443577	NM_014452	TNFRSF21	Tumor necrosis factor receptor superfamily, member 21
G02	Hs.462529	NM_003790	TNFRSF25	Tumor necrosis factor receptor superfamily, member 25
G03	Hs.654459	NM_001561	TNFRSF9	Tumor necrosis factor receptor superfamily, member 9
G04	Hs.478275	NM_003810	TNFSF10	Tumor necrosis factor (ligand) superfamily, member 10
G05	Hs.654445	NM_001244	TNFSF8	Tumor necrosis factor (ligand) superfamily, member 8
G06	Hs.654481	NM_000546	TP53	Tumor protein p53
G07	Hs.523968	NM_005426	TP53BP2	Tumor protein p53 binding protein, 2
G08	Hs.697294	NM_005427	TP73	Tumor protein p73
G09	Hs.460996	NM_003789	TRADD	TNFRSF1A-associated via death domain
G10	Hs.522506	NM_021138	TRAF2	TNF receptor-associated factor 2
G11	Hs.510528	NM_003300	TRAF3	TNF receptor-associated factor 3
G12	Hs.356076	NM_001167	XIAP	X-linked inhibitor of apoptosis
H01	Hs.520640	NM_001101	ACTB	Actin, beta
H02	Hs.534255	NM_004048	B2M	Beta-2-microglobulin
H03	Hs.592355	NM_002046	GAPDH	Glyceraldehyde-3-phosphate dehydrogenase
H04	Hs.412707	NM_000194	HPRT1	Hypoxanthine phosphoribosyltransferase 1
H05	Hs.546285	NM_001002	RPLP0	Ribosomal protein, large, P0
H06	N/A	SA_00105	HGDC	Human Genomic DNA Contamination
H07	N/A	SA_00104	RTC	Reverse Transcription Control
H08	N/A	SA_00104	RTC	Reverse Transcription Control
H09	N/A	SA_00104	RTC	Reverse Transcription Control
H10	N/A	SA_00103	PPC	Positive PCR Control
H11	N/A	SA_00103	PPC	Positive PCR Control
H12	N/A	SA_00103	PPC	Positive PCR Control

## Footnotes

1 National Cancer Institute (2024). Drugs Approved for Melanoma [online]. Website https://www.cancer.gov/about-cancer/treatment/drugs/melanoma [accessed 10 April 2024].
